# Philadelphia Correspondence

**Published:** 1868-04-01

**Authors:** E. K. Hutchins

**Affiliations:** Philadelphia, Penn.


### PHILADELPHIA CORRESPONDENCE.

PHILADELPHIA, PENN., *March 2nd*, 1868.

*Chicago Medical Journal:*

W. M., age 42 years, blacksmith, presented himself with a large tumor at the bend of the right elbow, measuring about — inches in length and the same in breadth, complaining of a dull heavy pain and numbness in the limb. This tumor was of nearly one year's growth. Ten days before its first appearance, he was kicked by a mule. The tumor was elastic, and harder at some points than at others. Preternatural redness and heat. There was a pulsation, which was referable to the displaced brachial artery. Patient was etherized, and an incision made, which revealed the hematoid variety of encephaloid tumor. The arm was amputated at shoulder joint. In four weeks, the patient was discharged, cured.

To-day closes the college for one month; and during the session just ended, we have had a large number of interesting cases for operation and treatment. I propose in this letter to give you a brief *resumè* of the surgical clinic, as it, like all others, presents valuable and interesting data. There have applied for surgical aid, *six hundred and forty-one persons.* Among this large number, let me enumerate the following: Amputations, five, consisting of one at the shoulder joint, for an encephaloid tumor, involving the elbow joint, one just above the knee, for inflammation of that joint, two of the index and one of the ring fingers; eight abscesses, acute and chronic, in various regions of the body; two cases of permanent anchylosis of the knee, and one of forefinger; two cases of cystitis; one of organic stricture of the urethra; two cases of hydrocele; two of varicocele; five cases of Potts' disease of the spine; atrophy of arm, from wound in hand; atrophy of leg, from wound of ankle; two cases of Barton's fracture; one fractured fibula; in another case, the fibula was intentionally broken, and the tendo-Achilles cut, to correct the deformity resulting from injury to ankle; intentional fracture was made of the olecranon, to place the forearm at right angles with the arm, in case of dislocation of three months' duration; dislocation of head of humerus into axilla; dislocation of head of radius; one case of perineal section, for rupture of neck of bladder; four cases of lithotomy, one on a boy four years of age, and one upon a man sixty-seven years old; two cases of necrosis of humerus, and one of necrosed tibia; a number of cases of syphilis, of all descriptions; one case of fissure of hard palate; four cases of fissured upper lip; neuralgia of inferior dental nerve, cured by trephining inferior maxillary bone, and removing 1¾ inches of the nerve. There have been seven cases of club foot, representing the varieties of talipes varus, talipes vulgus, and talipes equinus; one case of phymosis; one of anal fistule; one of ligature of femoral artery, for the radical cure of popliteal aneurism; two cases of sciatica; three of coxalgia, chronic ulceration of leg, treated by scarification; six cases of converging and diverging strabismus; two ulcerations of cornea; three of cataract; one of congenital blindness; one of deposit of lymph in anterior chamber of the eye; schirrus of mammary gland; cystic ditto; encephaloid ditto. Five fatty tumors were removed, from the size of fist to one ten pounds in weight; one immense fibroid tumor, from sacrum; one keloid, at base of ear; five encephaloid tumors; three parotid ditto; three cystic ditto; one reducible scrotal hernia. In addition to these, there have been many others of more or less importance. In all these cases, but two deaths have resulted; one from the operation of lithotomy, and the other in the case of perineal secture. Both are interesting cases, and in my next I shall give you a full account of them, with the autopsy of each.

Yours, E. K. Hutchins.

